# A toxicity-dependent feasibility bound for the escalation with overdose control approach in phase I cancer trials

**DOI:** 10.1186/1745-6215-16-S2-P233

**Published:** 2015-11-16

**Authors:** Graham Wheeler, Michael Sweeting, Adrian Mander

**Affiliations:** 1 MRC Biostatistics Unit Hub for Trials Methodology Research, Cambridge, Cambridgeshire, UK; 2 Strangeways Research Laboratory, University of Cambridge, Cambridge, Cambridgeshire, UK

## 

Phase I trials of anti-cancer therapies aim to identify a Maximum Tolerated Dose (MTD), defined as the dose that causes unacceptable toxicity in a target proportion of patients. In such trials, the dose given to patients is adapted as dose-response data are accrued and the next patient may receive a higher, lower, or identical dose to that of previous patients.

Both rule- and model-based methods have been proposed for conducting dose-escalation studies and recommending an MTD. The Escalation with Overdose Control (EWOC) approach is a model-based design where the dose assigned to the next patient is one that, given all available data, has a posterior probability of exceeding the MTD equal to a pre-specified value known as the feasibility bound. The aim is to conservatively dose-escalate and approach the MTD, avoiding severe overdosing early on in a trial. Methodological and applied research has considered the EWOC approach with the feasibility bound both fixed and varying throughout a trial, yet some of the methods may recommend incoherent dose-escalation; that is, the next patient may be recommend a higher dose even though patients being treated at the previous dose experienced severe toxicities.

To counter this, we propose a toxicity-dependent feasibility bound that guarantees coherent dose-escalation and incorporates the desirable features of other EWOC approaches. We show via detailed and comprehensive simulation studies that our approach provides improved MTD recommendation properties over the traditional EWOC approach, as well as comparable operating characteristics relative to modified EWOC approaches, whilst guaranteeing coherent dose-escalation.

